# Linking Household and Service Provisioning Assessments to Estimate a Metric of Effective Health Coverage: A Metric for Monitoring Universal Health Coverage

**DOI:** 10.3390/ijerph22040561

**Published:** 2025-04-03

**Authors:** Veenapani Rajeev Verma, Shyamkumar Sriram, Umakant Dash

**Affiliations:** 1 Department of Humanities and Social Sciences, Indian Institute of Technology Madras, Chennai 600036, India; veenapani.verma1@gmail.com; 2 College of Health and Public Service, University of North Texas, Denton, TX 76203, USA; 3 Institute of Rural Management Anand, Gujrat 388001, India; dash@iitm.ac.in

**Keywords:** effective health coverage, universal health coverage, multidimensional composite metric, fragile setting, geospatial linking techniques, inequities in effective coverage, quality of care

## Abstract

Background: The framework of measuring effective coverage is conceptually straightforward, yet translation into a single metric is quite intractable. An estimation of a metric linking need, access, utilization, and service quality is imperative for measuring the progress towards Universal Health Coverage. A coverage metric obtained from a household survey alone is not succinct as it only captures the service contact which cannot be considered as actual service delivery as it ignores the comprehensive assessment of provider–client interaction. The study was thus conducted to estimate a one-composite metric of effective coverage by linking varied datasets. Methods: The study was conducted in a rural, remote, and fragile setting in India. Tools encompassing a household survey, health facility assessment, and patient exit survey were administered to ascertain measures of contact coverage and quality. A gamut of techniques linking the varied surveys were employed such as (a) exact match linking and (b) ecological linking using GIS approaches via administrative boundaries, Euclidean buffers, travel time grid, and Kernel density estimates. A composite metric of effective coverage was estimated using linked datasets, adjusting for structural and process quality estimates. Further, the horizontal inequities in effective coverage were computed using Erreygers’ concentration index. The concordance between linkage approaches were examined using Wald tests and Lin’s concordance correlation. Results: A significantly steep decline in measurement estimates was found from crude coverage to effective coverage for an entire slew of linking approaches. The drop was more exacerbated for structural-quality-adjusted measures vis-à-vis process-quality-adjusted measures. Overall, the estimates for effective coverage and inequity-adjusted effective coverage were 36.4% and 33.3%, respectively. The composite metric of effective coverage was lowest for postnatal care (10.1%) and highest for immunization care (78.7%). A significant absolute deflection ranging from −2.1 to −5.5 for structural quality and −1.9 to −8.9 for process quality was exhibited between exact match linking and ecological linking. Conclusions: Poor quality of care was divulged as a major factor of decline in coverage. Policy recommendations such as bolstering the quality via the effective implementation of government flagship programs along with initiatives such as integrated incentive schemes to attract and retain workforce and community-based monitoring are suggested.

## 1. Background

The concept of effective coverage refers to the proportion of potential health benefits that the population actually receives through the health system, given its capacity. It consists of three elements: need, use, and quality. Need refers to the individual/population in need of a particular service, use refers to the use of services, and quality refers to the actual health benefit experienced from the service [1]. The metric of effective coverage can be aggregated across a large, diverse set of interventions to proxy the effectiveness of an entire healthcare system. This concept of effective coverage is conceptually straightforward, yet translation into single metric is quite intractable. An estimation of metric linking need, access, utilization, and service quality is imperative for measuring progress towards universal health coverage. Further, the estimation of effective coverage at a subnational level in India is paramount as the dissimilitude in the state’s epidemiology, health system financing, and level of socioeconomic development purports both different approaches to universal health coverage as well as a potential range of relevant metrics. Cultures and geography impact intervention designs and can thus help identify the implementation bottlenecks in a more nuanced way, remove ecological bias, and thus obtain subnational level estimates that are particularly important when effective coverage is used to monitor progress towards universal health coverage [1].

The Tanahashi framework [2] proposed a cascade approach that defines and organizes the components of coverage in a stepwise fashion, aiding in the identification and quantification of the losses to the potential health benefits declining from one stage to another. The slump from crude/contact coverage to effective coverage is the final hypothetical stage representing a cascade of the loss in coverage due to the quality of care and is representative of the bottleneck in effectiveness. Health service quality encompasses a multitude of dimensions including patient safety, effectiveness, people centredness, and integratedness [3]. Donabedian classified the measures to assess and compare the quality of healthcare organizations into structure (service availability and provider readiness measures), process (adherence to protocols and standards of care by providers), and outcome measures (health benefits and patient’s satisfaction) [4]. There are a battery of studies employing structural measures of quality in the estimation of effective coverage. However, process and outcome measures of quality indicating person-centred care are under-represented in the literature. The coverage metric obtained from a household survey is not succinct as it only captures service contact that cannot be construed as actual service delivery and ignores the comprehensive assessment of provider–client interaction and quality of services. As contact coverage does not necessarily translate to effective coverage, it is pertinent to measure the quality-coverage gap.

In the Indian context, specifically, the evaluation of healthcare interventions and reforms has invariably faced challenges because of the lack of reliable administrative data [5]. Recent studies in India divulge that measurement methods linking the content (quality) to the contact (utilization) result in considerably lower quality contacts than crude contact estimates [6,7]. Further, the quality of services has also been revealed to be a major reason for the higher unmet need for treatment seeking from public health facilities in India [8]. The evidence on quality-adjusted coverage from India also suggests pervasive socio-economic heterogeneities, indicative of the significant inequities for economically and socially vulnerable populations [7,9]. However, the collection and monitoring of quality indicators are either missing or are scant in the Indian setting. On one side, Service Provisioning Assessment (SPA)/health facility surveys collect data on just the service availability and facility’s readiness to provide a particular service. On the other hand, population surveys such as national sample surveys (NSSs) and the National Family Health Survey (NFHS) focus on capturing the information on the population’s healthcare needs and population-based health outcomes such as contact coverage/utilization of an intervention. However, a linked analysis of population surveys and facility assessment enables examination between population healthcare needs, their contact coverage, and the health service environment [10]. Thus, it is imperative to conflagrate information on access, utilization, and the quality of health services by linking temporally and geographically proximate household and healthcare provider data.

The operationalization of linking of these datasets can be conducted in a multitude of ways ranging from exact-match linking, to capturing the service environment via ecological linking using geospatial techniques. In low-income and middle-income countries (LMICs), including India, the ideal method for coalescing household and health provider data is at the individual level, or exact-match linking, where information for each care-seeking episode (i.e., every time an individual in need of care seeks care) recorded in a household survey is linked to the information about the quality of care of the specific provider(s) visited during that episode. However, this approach is not considered feasible for routine monitoring via national sample surveys in most LMICs because of the reasons such as: (a) difficulty in obtaining an accurate and comprehensive list of providers that can be integrated into household surveys, (b) impediments in eliciting the source of care from respondents, and (c) resources required to collect data on all the providers potentially visited, especially informal providers [2].

Few studies [11] have been conducted to establish the link between individual health facilities and survey respondents; however, previous studies have several lacunae with respect to methodological issues. Firstly, most of the Service Provisioning Assessments are surveys which are not representative at the disaggregated geographical levels; thus, they are susceptible to misclassification errors and are constrained in their representativeness. Secondly, the ecological linking via GIS-techniques such as raster-based methods and Kernel density estimates (KDEs), which accounts for physical barriers, facility characteristics, and population draw towards facilities has not been explored much in the literature. There are a few studies that have used the KDE-based linking of surveys, but they lack methodological rigour due to the strong constraining assumptions pertaining to the parameter bandwidth, rate of distance–decay function, and exclusion of physical barriers in the estimation of the KDE function. Thirdly, the few studies investigating quality-adjusted coverage are limited to only the objective criterion of structural quality, notwithstanding the subjective criterion of patients’ perception of care and process quality measures. Fourthly, the previous literature has precluded the adjustments for inequity in the quality-adjusted measure of coverage. Our study, hence, strived to bridge these methodological gaps in the literature. The novelty of our study stems from linking the various datasets (i.e., household survey, health provider assessment, and patient exit interviews) using geospatial techniques to coalesce the measures of need, utilization, and quality of care.

Given the backdrop, our study espouses the following objectives:

(a) Link the household survey, provider assessment, and patient exit survey using exact-match and ecological-linking techniques.
(b) Estimate a metric of effective coverage of healthcare interventions encompassing antenatal, delivery, postnatal, immunization, ambulatory, and inpatient care in the study area.
(c) Compare the effective coverage estimates obtained using exact-match and ecological linking methods.

## 2. Methodology

### 2.1. Area Setting

The study was conducted in Poonch district which is the remotest district in the Union Territory of Jammu and Kashmir in India. The region is the northernmost part of India with a hilly and mountainous topography and the Himalayan range of Pir Panjal separating the district from the Kashmir region. The altitude of the area varies from 1007 m to 4700 m above sea level and is susceptible to inclement weather conditions with snow-cover during winters. The district has a rural population of 438,205 dispersed over 178 villages and urban population of 38,630 (concentrated in the district centre). The entire district is 1674 square kilometres with a population density of 285 per square kilometre. The region has a literacy rate of 68.60% with more than one-third of the population belonging to the socially disadvantaged groups. Also, the district is relatively impoverished with one-third of the population living below the poverty line.

The district is low performing in terms of health indicators and has been categorized as a high priority district by the Government of India based on the poor performance in the health composite index. The district is divided into three medical blocks circumscribing the medical infrastructure of (a) 1 district hospital and 3 community health centres (DH and CHCs) providing secondary healthcare, (b) 44 primary health centres (PHCs) serving as the first point of contact between the population and doctors, and (c) 137 subcentres which are the peripheral health institutions available to rural population serving as the first point of contact with healthcare providers. There are insurmountable healthcare access barriers in the region translating into suboptimal effective coverage which is exacerbated by the conflict in the region. The district is a conflicted borderland with heavy military deployment and armed militancy as it is bounded by a Line of Control (porous boundary between Indian and Pakistani-administered Kashmir and is one of the most complex frontier systems in the world) in the western part. The border villages are assailable to firing and shelling due to the porous borders which are fenced and at numerous places gated, enclosing swathes of population with ensuing obstructions to access.

### 2.2. Study Design, Participants, and Data Collection

Three distinct tools were employed in the study to estimate the metric of quality adjusted coverage, i.e., (a) household/population health survey (b) health facility assessment and (c) patient exit survey. The survey data for the study were collected during the period of March to October 2019. The details of the tools used are explicated in the section below.

#### 2.2.1. Household Survey

A district representative household survey employing two-stage stratified random sampling was conducted to elicit information on crude coverage/care seeking for varied dimensions of care. Crude coverage is operationally defined as the proportion of individuals in need of a specific health service who report having received it, regardless of the quality or appropriateness of care delivered. It captures utilization alone and serves as a basic measure of access to healthcare services [1].

A list of villages in the district from Socio-Economic Caste Census 2011 was obtained and arranged in ascending order of population which was classified into four stratums viz. Stratum 1—villages having a population of within 1000 individuals, Stratum 2—villages with 1000–2000 individuals, Stratum 3—villages with 2000–5000 individuals, and Stratum 4—villages exceeding 5000 individuals. In the first stage, villages were selected as first-stage units (FSUs) randomly within the four stratums as per population proportionate to size resulting in the selection of 15, 11, and 14 villages in Mandi, Surankote, and Mendhar, respectively. In the second stage, households were selected randomly using methodology devised by the National Sample Survey Organization for Health and Morbidity Survey. A total of 200 households per selected village were screened and subsequently, 40 households were randomly selected in the ratio of 16:8:8:8 for inpatient cases, ambulatory cases, pregnant cases, and immunization, respectively in each village culminating in a total sample size of 1600 households (600, 440, and 560 households from Mandi, Surankote, and Mendhar, respectively) enclosing 589 ambulatory, 640 inpatient, 320 maternal care, and 780 immunization care-seeking episodes. The geo-coordinates of sampled households were collected using GPS device Garmin eTrex10 (Garmin, Chennai, India).

A close-ended questionnaire was designed for collecting information pertaining to households’ and individuals’ socio-economic background, morbidity status, utilization of healthcare services, and healthcare expenditure. The details of service coverage indicators, target population, and definitions are summarized in the Supplementary File: Table S1. Crude coverage estimates of care-seeking episodes sought in public health facilities were generated on the scale of 0–100% for each service package, i.e., antenatal care, delivery care, postnatal care, immunization care, ambulatory care, and inpatient care. Further, we estimated the horizontal inequity indices using Erreygers’ concentration index and computed inequity-adjusted crude coverage for various service packages. The estimates for crude coverage and inequity-adjusted coverage were weighted using sample weights.

#### 2.2.2. Health Facility Assessment

A Master Facility list consisting of administrative information on facility type, name, and codes (identifier used in a health management information system), administrative units, location/address, and contact information was created. A census of public health facilities including district hospital (*n* = 1), community health centres (*n* = 3), primary health centres (*n* = 17), new-type primary health centres (*n* = 27) and subcentres (*n* = 137) was conducted to discern the readiness/structural quality of facilities. The geo-coordinates of the sampled households were collected using the GPS device, Garmin eTrex10. A tool was modelled based on the Service Availability and Readiness Assessment (SARA) M&E tool which was designed by the World Health Organization in conjunction with the United States Agency for International Development (USAID) for systematic facility surveys and was used in tandem with the Indian Public Health Standards (IPHS).

Information on service-specific readiness connoting the ability of health facilities to offer a specific service and readiness to provide that service was ascertained through a set of tracer indicators subsumed under major domains such as: (a) physical infrastructure and amenities, (b) human resources, (c) equipment, (d) drugs and consumables, and (e) infection control protocol. A compendium of checklists was designed in the form of a standard core questionnaire gauging the availability of tracer indicators, and a scorecard was generated for each facility. Data were coded into binary with 1 indicating that an item was observed and present and 0 indicating that an item was not observed and present. Service-specific readiness of the facility was derived by computing the mean availability of tracer items for each subdomain viz. physical infrastructure and amenities, human resources, equipment, drugs and consumables, and infection control protocol across each facility. The illustrative list of tracer items used in the construction of structural quality scores is delineated in Table S2.

This measurement of service readiness corresponds to the operational definition of the structural quality of care, which encompasses material and organizational components—such as infrastructure, equipment, supplies, and staffing—necessary to deliver healthcare services [12]. Structural quality represents a facility’s capacity to provide services and is a critical precondition for ensuring safe and effective care [13,14].

#### 2.2.3. Patient Exit Interviews

A patient exit survey encompassing a sample size of 850 clients (*n* = 50 for each level of facility and service type) was conducted to unravel the process quality of care. A Patient Satisfaction Questionnaire (PSQ) was administered amongst the patients exiting the public health facility after seeking care to evaluate the relationship of a client/patient’s attitude pertaining to the personal and practice characteristics of providers. A multidimensional construct reflecting patients’ expectations, values, and experiences was constructed eliciting information on various domains of perceived quality of care such as Interpersonal relationships, Affordability, Accessibility, Technical Quality, Physical Environment, Efficacy and Continuity.

An amalgamation of categorical response choices and Likert-type items were used as tracer indicators across the domains of Interpersonal Components, Affordability Components, Accessibility Components, Availability Components, Technical Quality Components, Physical Environment Components, Efficacy Components, and Continuity Components (details on Table S3). The components were coalesced via Factor Analysis to compute scores across domains and to compute the overall service-specific composite score for, antenatal care, delivery care, post-natal care, immunization care, ambulatory care and inpatient care. The construction was symptomatic to process the quality of care and accommodation, i.e., the way supply-side resources are organized to accept a client’s preferences and abilities to accommodate these factors and their perception of what is appropriate.

This approach reflects the operational definition of process quality of care, which refers to the extent to which care delivery aligns with accepted clinical standards and best practices during the interaction between provider and patient. It encompasses not only technical competence but also interpersonal and communication quality, adherence to guidelines, and responsiveness to patient needs and preferences [13,15].

### 2.3. Linking

**Figure 1:**
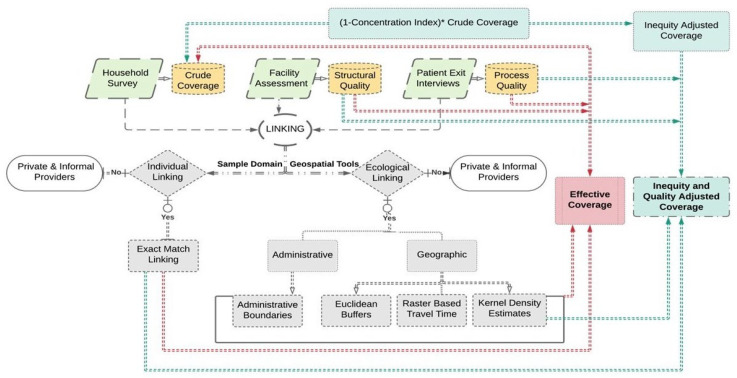
Schema of linking varied surveys to estimate effective coverage.

Multifarious linking methods encompassing both individual and ecological approaches were applied to conjoin data on population outcomes with the relevant service environment. The coverage estimates are sensitive to the choice of approach and type of quality measure to link household and provider data. A detailed scheme of approaches which was employed to link datasets to compute the effective coverage are represented in Figure 1.

The individuals/episodes who sought care from public providers were included in the study, and those who sought care from private/informal providers were excluded. Separate linkages were mapped for a varied package of services by assigning both structural and process quality scores to contact coverage. The linking methods employed in the study are described as follows:

#### 2.3.1. Individual Linking (Exact Match Linking)

Exact-match linking assigned provider information to care-seeking episodes experienced by the target population based on their specific source of care. Each care-seeking episode for maternal (ANC, delivery, and PNC), immunization, and ambulatory and inpatient care were assigned the structural quality score of healthcare facility from where care was sought.

An average of process quality score for the type of provider was also assigned to care-seeking episodes. A score of zero was attributed to episodes for which care was sought from public health facilities outside the district; however, if care was sought from more than one provider, an average score of providers was assigned to the episode. The proportion of care-seeking episodes linked via exact-match linking is presented in Table 1. This approach, albeit considered as the most precise method for linking datasets at a disaggregated level, is susceptible to reporting bias and self-selection bias [16]. Thus, an alternative approach of ecological linking was employed to link the datasets, as below:

#### 2.3.2. Ecological Linking

Ecological linking assigned the provider information to care-seeking episodes based on the geographic criterion. The characteristics and quality of providers from where the care is sought is disparate from the providers where care is not sought, transmuting into an over-estimation of the effective coverage. Therefore, an alternative approach capturing the service environment with distinct spatial joins was also explored in the study. The ecological approaches employed in this study are elucidated as below:

##### Linking via Administrative Unit: HFCA

**Figure 2:**
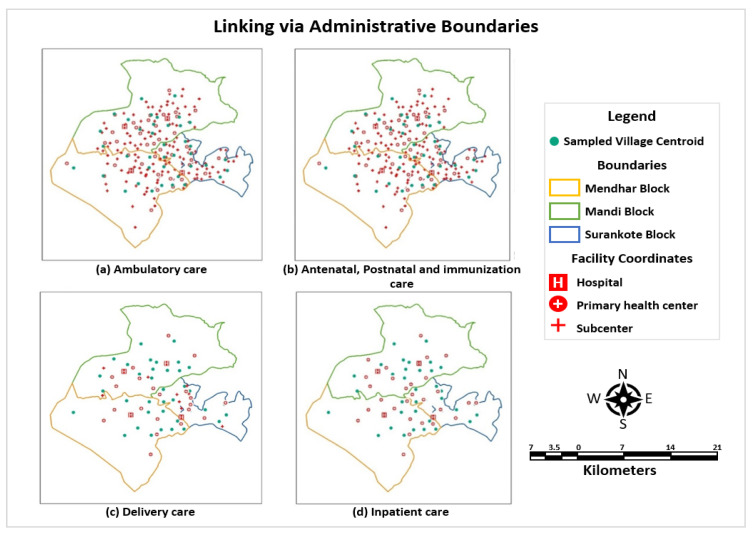
Depiction of ecological linking via administrative boundaries.

This method is underscored by the assumption that the overall service environment in an administrative area is representative of the service environment for any FSU/Hamlet. As represented in Figure 2, each care-seeking episode was linked to all the providers within the category of source and package of care. The average structural and process quality scores of all providers within each medical block were assigned to the household within the reported category of source of care (subcentre, PHC, CHC, and DH). Linking via administrative boundaries is sensitive to high ecological bias as each village centroid is linked to every single facility within an administrative unit, creating a homogenous service environment with an administrative boundary [17]. Henceforth, other approaches delving into geospatial linking were also performed to discern the metric of effective coverage.

##### Geographical Linking

Geographical linking employed a suite of Geographic Information System (GIS) techniques using both raster and vector-based methods to link geographically concordant household and provider data to develop the indices of effective coverage. It is a relatively more rigorous and realistic ecological method that employs geographic boundaries to encapsulate the service environment instead of administrative boundaries. Three distinct methods of linking via geographical boundaries were implemented, adopted from Skiles et al. [18] and Carter et al. [19] which are summarized below:

(a) Linking via Euclidean Buffers

**Figure 3:**
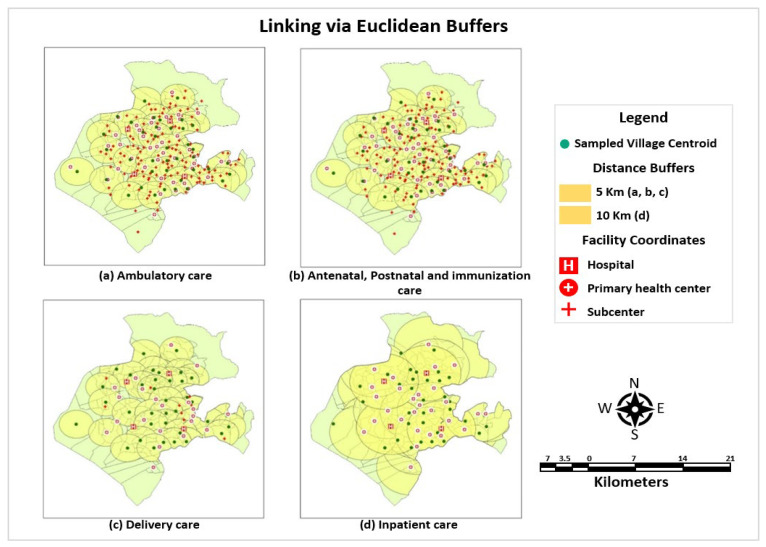
Depiction of geographical linking via Euclidean buffers.

The distance (Euclidean) buffers were created using a Feature-based Proximity tool where vector polygons at a specified buffer distance enclosing the spatial location of village centroids as the input features. In tandem with the local settings, a buffer size circumscribing 5 km radii for ambulatory, immunization, and maternal care and 10 km for inpatient care were generated. The features were dissolved to eliminate the overlapped areas circumventing the double count. A point layer of facilities was overlaid on village boundaries’ shapefile, and spatial joining was performed to discern the availability of facilities delivering the specific service within the buffer zone. A map depicting this linking approach is illustrated in Figure 3.

An average of structural and process quality score of facilities providing service within the buffer zone was assigned to the households. However, buffers may overestimate or underestimate the population inside the defined buffers; hence, more rigorous raster-based geospatial techniques were also employed in the study.

(b) Linking via Raster-Based Travel Time

**Figure 4:**
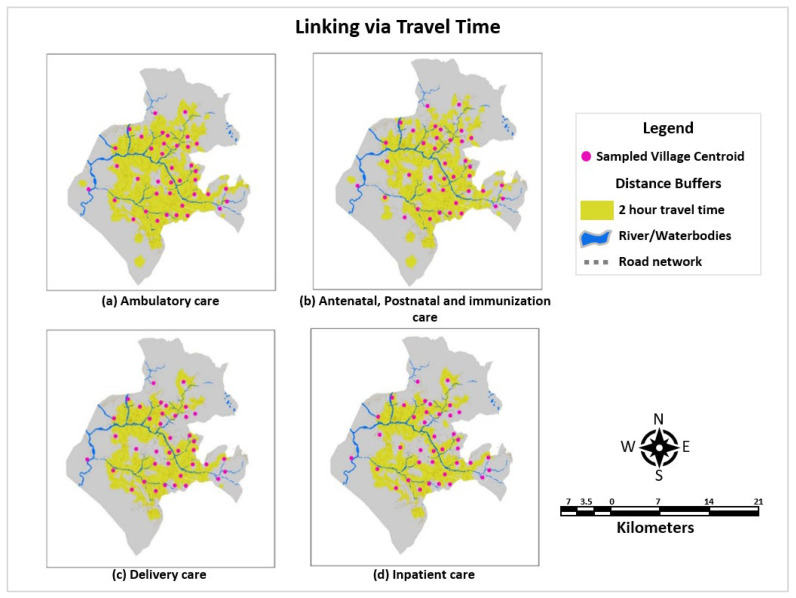
Depiction of geographical linking via travel time.

A more robust approach of the least cost path (friction surface) to produce a raster layer across the target area was adopted to link travel time grids with the population data. A raster surface of the travel time between facilities and population was developed by incorporating the terrain, physical barriers, topography, and travelling modes through various land-cover classes as shown in Figure 4. The inputs, parameters, spatial modelling approach, and output presenting a travel time distribution grid for all packages of services were explained in our previous work [20]. A travel time distribution grid of 2 h with the highest proportion of travel scenarios sought by aiming for a particular package of care (walking only for ambulatory and immunization care, walking and private/rental vehicle for delivery and inpatient care, walking and public transportation for antenatal care and postnatal care) was selected to link the survey data.

An average structural and process quality score of all the providers within a 2 h travel impedance was linked to households with care-seeking episodes. However, such mapping and linking approach precluded the provider’s characteristics and distance decay assumption (draw of the facility from the population decreases as the distance from the facility increases in determining the pull exerted by the facilities). These characteristics were thus incorporated into our methods using the Kernel density estimates approach.

(c) Linking via Kernel Density Estimates

A computationally intensive non-parametric approach of kernel density estimation (KDE) was employed to link the health service environment characteristics with the surveyed households. KDE is more robust than other methods as the following characteristics are embedded in this approach: (a) providing an estimate of density in a continuous surface which is unrestrained by administrative boundaries, and thus is a better representation of the spread of people and services across a landscape, (b) embodying a decaying effect of distance on a facility’s service area, and (c) examining an additive service environment with the conjecture that a high service environment from multiple facilities has a greater effect than a high service environment from a single facility [21].

Through this approach, the weighted pull of the providers was accounted for to reflect the level of draw which health facilities exert over the households within their catchment area as a source of care for patients. Using the distance decay function, the KDE accounted for the pressure on treatment seeking that emanated from the characteristics of providers such as provider type, distance to provider, and provider readiness and preparedness.

The weighting of distance of care-seeking episodes from a particular provider, *x*, can be expressed mathematically as follows [22] (Equation (1)):

(1) $$f\left(x\right)=\frac{1}{nh}\;\sum_{i=1}^{n}k\left(\frac{x-x_{i}}{h}\right)$$

where *n* is the number of observations in the sample, *h* is the bandwidth or the smoothing parameter, *k*(*x*) is the defined kernel function, and (*x* − *xi*) represents the Euclidean distance between each point *i* and the provider location. The kernel bandwidth/search radius (*h*) can be computed as (Equation (2)):

(2) $$Search\;Radius=0.9\times min\left(SD,\sqrt{\frac{1}{ln(2)}\times D_{m}}\;\right)\times n^{-0.2}$$

where *Dm* is the (weighted) median distance from the (weighted) mean centre, *n* is the number of points if no population field is used, or if the population field is supplied, *n* is the sum of population field values, and *SD* is the standard distance.

Parameters such as kernel bandwidth/kernel size, probability density distribution function, and grid size were specified to link the datasets via KDE (the details are in the Supplementary File). Most of the previous studies assign arbitrary bandwidths as parameters, which requires strong assumptions pertaining to the rate at which health facility readiness decays over space. However, in our study, distinct bandwidths (catchment sizes) were defined by taking the cognizance of the preference of the secondary and tertiary providers by users. The provider quality score (structural and process) was specified as the density/population value, modelling stronger pull/draw within their catchment area for providers with better quality scores. Gaussian/normal distribution assigning greater weight closer to the centre and lesser to the parameters was used in the study. Finally, a grid size of 100 m was set to extricate the output raster. To improve the methodology for estimating a more realistic and accurate surface, KDE incorporating physical barriers such as waterbodies and snow-capped areas, etc. were employed in this study.

**Figure 5:**
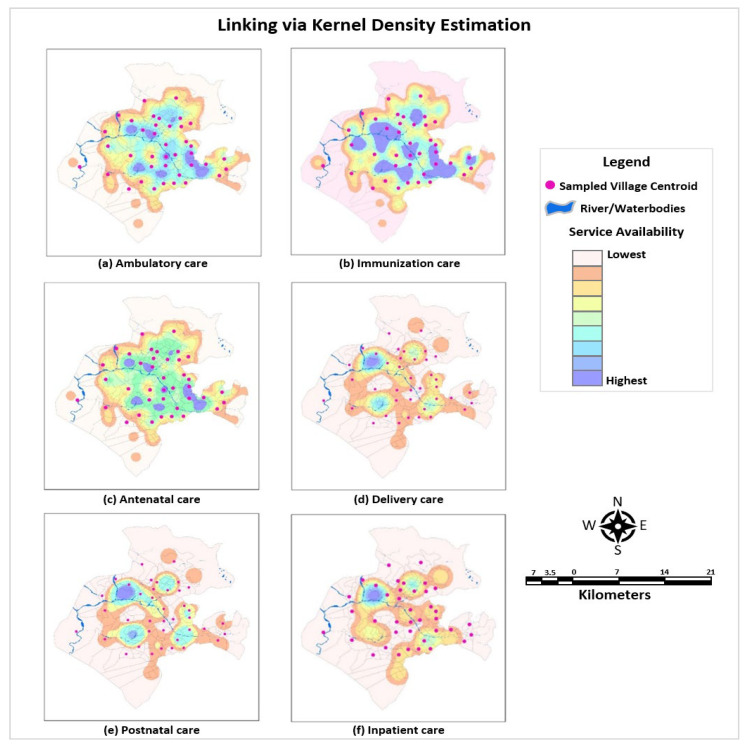
Depiction of geographical linking via kernel density estimation.

Operationalization of linking via KDE is illustrated in Figure 5 which encompasses the assignment of distance-weighted facility readiness estimates to the sampled households. Firstly, the KDE for each facility type was created separately and then summed within each grid cell using a raster calculator tool, allowing the execution of Map Algebra to create the KDE total layer. Figure 5a–f represent the projected map surface, visually describing the estimated influence of a given facility over space. Secondly, the overlapping catchment areas were summed for FSU/villages which were located near multiple facilities, thereby providing a better representation of the total service environment. Thirdly, extracting value to points function was used to compute the raster value at point locations of each household, and buffer extraction was used to determine the average value around village centroids. Fourthly, the care-seeking episodes were linked to the closest provider providing specific services and exerting any pull on the household. The structural and process quality scores assigned to each care-seeking episode were weighted based on the level of draw exerted by the provider. Hence, KDE linking via weighted pull was conducted; it examined an additive service environment with an assumption that a high service environment from multiple facilities has a greater effect than a high service environment from a single facility. The estimated surface areas were modelled via ArcGIS PRO with the optional ‘barrier parameter’ in the kernel density tool. The attribute tables for proximity measures generated in ArcGIS were exported to Stata and merged with the household survey data on contact/crude coverage.

### 2.4. Estimation of the Effective Coverage Metric

The metric of effective health coverage was computed by adjusting the use of the healthcare/crude coverage of services by the population in need for the quality of the intervention received by the population. The crude/contact coverage estimates were multiplied by average structural and process quality scores (according to the type of linking) to yield the effective coverage, represented as follows (Equation 3):

(3) EC=CC×Q

where EC represents the effective coverage, CC denotes crude or contact coverage and Q signifies the average structural and process quality score of the healthcare facilities (according to the type of linking).

The estimates were weighted by survey weights, and the analysis was conducted using STATA 14.2 (College Station, TX, USA). Since the census of public health facilities was conducted, it was assumed that there was no sampling error. The absolute and relative differences between ecological and exact-match estimates for each type of service was examined, and Wald tests were used to assess whether the differences between the estimates were statistically significant. The agreement between linking methods was also examined using Lin’s concordance correlation coefficient, and the Bradley–Blackwell F-test was used to test the statistical significance of concordance.

## 3. Results

### 3.1. Proportion of the Episodes Linked

The proportion of care-seeking episodes linked to any public provider by service and linkage type is illustrated in Table 1. The proportion of linkage via the exact-match linking approach was the highest for immunization care (100%) and antenatal care (100%) as the entire population in need (children up to 2 years of age and pregnant women) who sought care for these services from public health facilities within the district only, rendering it feasible to link all the episodes with the exact source of care as a census of public health facilities was conducted. There was a slight decline in linking percentage via this approach for delivery and postnatal care as few pregnant women sought these services from public health facilities outside the district, and thus were not under the purview of assessment. Analogously, the proportion of linking for ambulatory care and inpatient care also witnessed some omissions on account of the exclusion of care seeking from facilities outside the study area.

Further, all the care-seeking episodes could be linked ecologically by adopting an administrative boundaries approach; however, the overall proportion of episodes linked using a 5 km buffer was reduced to 91% for immunization, 88% for ambulatory and antenatal care, 86% for postnatal care, and the lowest of 75% for delivery care. Correspondingly, with a 10 km buffer for inpatient care, 86% of hospitalization spells were linked. The relatively low level of linking was attributed to the spatial inaccessibility of the households within the buffer zone. The proportion of linking further plummeted with linking via the raster-based travel time approach as a significant number of sampled households were geographically inaccessible from health facilities and were beyond the 2 h specified travel time grid. Only 40% of immunization cases, 39% of ambulatory, antenatal and postnatal cases, and less than one-third of cases for delivery (30%) and inpatient care (28%) could be linked to the facilities using the travel time grid. Similarly, fewer care-seeking episodes were linked to at least one provider via weighted kernel density estimation approach due to the low density of providers and quality score and thus lower weight exerted by providers. Care-seeking episodes which could not be linked were assigned average structural and process quality scores for the provider category of source of care.

### 3.2. Estimates of Composite Scores

The estimates for crude/contact coverage, structural quality, and process quality are summarized in this section succinctly. As illustrated in Table 2, there was a marked dissimilitude in the utilization of public health facilities by the type of service and level of provider.

#### 3.2.1. Crude Coverage

For routine immunization, 94.5% children were vaccinated in the public health facilities with the highest percentage of contact in peripheral facilities, i.e., subcentres (61.6%). The subcentres also exhibited horizontal equity in the coverage of immunization care (HI = 0.00); however, 15.4% and 14.8% of children received immunization in hospitals and primary health centres with marginal pro-poor inequities. Contrarily, coverage was acutely low for ambulatory care with only 34% ailing episodes (19.0%, 8%, and 7% in hospitals, primary health centres and subcentres, respectively) treated in public health facilities, which were additionally entrenched in inequities (HI = 0.09, 0.03, and 0.02 in hospitals, primary health centres and subcentres, respectively), translating into a further drop in inequity-adjusted estimates. Correspondingly, the utilization of facilities for antenatal care also slumped with only 18.9%, 8.6%, and 8.8% of ANC visits being conducted at hospitals, primary health centres, and subcentres, respectively with utilization concentrated amongst the poor (HI = 0.07) in the subcentres. In congruence with the immunization care, the contact coverage of delivery care was also perceptibly high in public health facilities with hospitals amounting to a disproportionately higher proportion (71.2%) of deliveries. The delivery-related episodes catered for in the hospitals were, however, inequitable, being concentrated amongst the rich (HI = 0.12); furthermore, only one-tenth of total deliveries were conducted in primary health centres and subcentres with concentration amongst the poor (HI = −0.02 and HI = −0.04, respectively). The contact coverage of postnatal care was found to be the lowest amongst all service types as only 8.5%, 5.6%, and 4.3% of mothers attended hospitals, primary health centres, and subcentres, respectively for postnatal visits with a high gradient between rich and poor (HI = 0.17 in hospitals, HI = 0.09 in primary health centres, and HI = −0.05 in subcentres). For inpatient care, 70% of hospitalization spells were treated in the hospitals, whereas 8.9% were treated in primary health centres with a relatively high gradient between rich and poor in hospitals (0.23) vis-à-vis primary health centres (0.04).

#### 3.2.2. Structural Quality

**Figure 6:**
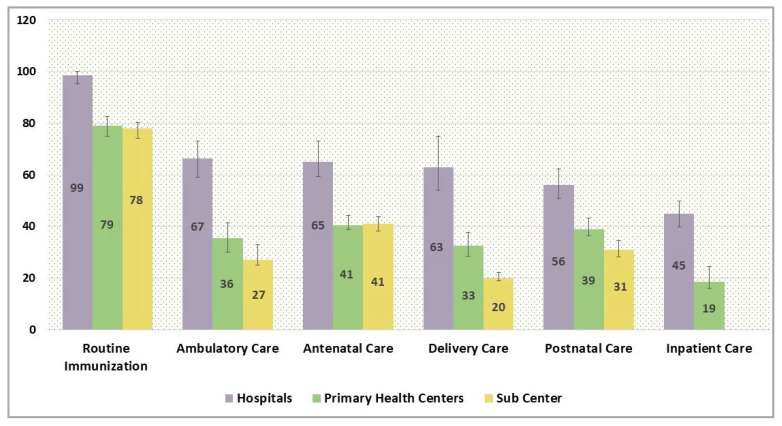
Mean readiness (structural-quality) score across packages of services.

Figure 6 expounds the structural quality score of the providers enunciating the readiness to provide care across packages of service. Pervasive heterogeneity was found in the quality of care between the level of facilities with higher-order facilities exhibiting higher scores. In order to manage immunization care, hospitals were accoutered with perfect readiness (99%), whereas primary health centres and subcentres had mean availabilities of 79% and 78%, respectively, to deliver routine immunization care. The readiness to manage general ambulatory care, however, was relatively poor across the facility type with hospitals being relatively more equipped (67%) to manage the ambulatory cases vis-à-vis primary health centres (36%) and subcentres (27%). Similarly, hospitals had 67% of recommended tracer items and staff to provide antenatal care, whereas primary health centres and subcentres possessed less than half (41%) of the resources for effective antenatal check-ups. The dissimilitude in the readiness of hospitals and lower-tier facilities for delivery care was further extensive with hospitals having 63% capacity as against the norms to manage the delivery cases vis-à-vis primary health centres that only had one-third of the resources with subcentres having just 20% of the recommended availability of resources. The structural quality for provisioning of postnatal care was also poor with mean availability scores of 56%, 39%, and 31% for hospitals, primary health centres, and subcentres, respectively. Furthermore, the readiness for the management of inpatient care was also inadequate with just a 45% score for availability of tracer indicators in hospitals and an abysmal 19% mean availability in primary health centres.

#### 3.2.3. Process Quality

**Figure 7:**
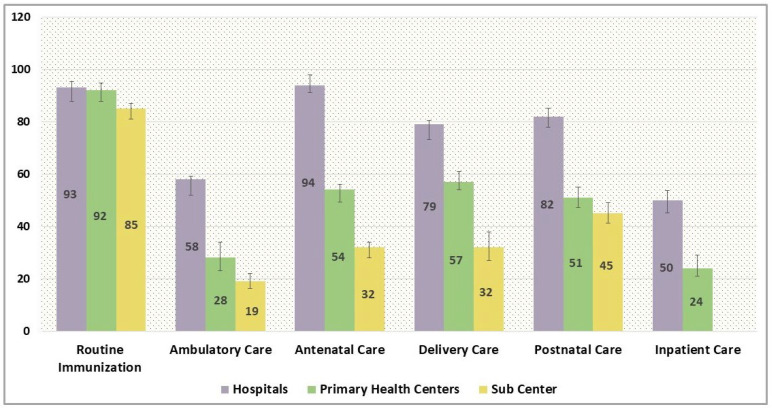
Mean process-quality score across packages of services.

The estimates of process quality as discerned through the patient exit survey are represented in Figure 7. The pattern and variations in process quality score across the services and facilities were cognate with the structural quality scores. The process quality for immunization care was the highest with a mean of 93% for hospitals and subsequently, 92% for primary health centres and 85% for subcentres. The patients’ overall satisfaction and perception of care regarding ambulatory services was however dampened with a mean score of 58% for the provisioning of outpatient care in hospitals and an average of 28% and 19% overall composite score for primary health centres and subcentres, respectively. For the antenatal visits, the process quality was comparatively high in hospitals (94%) vis-à-vis primary health centres and subcentres. Consonantly, patients exiting the hospitals post-delivery attributed a higher score of 79% in hospitals, as compared to 57% in primary health centres and subcentres, respectively. The patient satisfaction for postnatal care ranged from 82% in hospitals to a low of 45% in subcentres. Amongst the service packages, patient satisfaction was the lowest for inpatient care with a process quality score of just half (50%) in hospitals, which was further contracted to 24% for primary health centres.

### 3.3. Estimates of Effective Coverage

The estimates of quality-adjusted, inequity-adjusted and both quality- and inequity-adjusted coverage estimates via the varied linking methods are represented in Table 3. The results across all the types of services and linking methods divulged a decrease in coverage levels when adjusted for structural/process quality of care. The estimates of effective coverage were hence, subjacent to the crude-coverage levels, thereby ascertaining the quality of care and supply-side characteristics of providers as a barrier towards achieving universal health coverage. The detailed results and discordance amongst the estimates derived via different linking methods are summarized in this section.

#### 3.3.1. Full Immunization

According to the household survey, a high proportion of children [94.5%, 95% CI: 89.6–98.2%] received full immunization in public health facilities, which declined to 75.9% [95% CI: 70.3–80.4] when adjusted for structural quality and exhibited a drop of 12.9% post adjustment with process quality when care-seeking episodes were linked via the exact-match approach. Similarly, inequity-adjusted coverage also plummeted by 17.1% and 11.5% after discounting for structural and process quality. The divergence between contact coverage and both structural- and process-quality-adjusted coverage increased marginally when linked via administrative boundaries vis-à-vis exact-match linking. Correspondingly, inequity-adjusted coverage also slumped by 19.6% and 13.5% with structural and process quality adjustment.

Further, linking via Euclidean buffers of 5 kms culminated in structural-adjusted coverage of 75% [95% CI: 70.3–78.7%] and process-adjusted coverage of 78.2% [95% CI: 75.3–82.5%]. The linking via a raster-based travel time grid of 2 h resulted in the shrink in effective coverage by 23.4% for structural quality adjustment and 16.5% for accommodation in process quality. The inequity-adjusted coverage also dwindled significantly after quality adjustments. Albeit that the drop in coverage with structural quality adjustment was the highest with linking via KDE as the coverage reduced to 70.4% [95% CI: 66.2–73.8%], the process-quality-adjusted coverage estimates were relatively higher at 78% [95% CI: 74.2–83.4].

#### 3.3.2. Ambulatory Care

For ambulatory care, only 33.9% [95% CI: 30.2–38.4%] ailment episodes were treated in the public health facilities, which further had pro-rich inequities, thereby resulting in even lower estimates for inequity-adjusted coverage. The poor readiness of healthcare facilities in providing ambulatory care resulted in the estimates of effective coverage being only half compared to crude coverage via all the linking approaches. For exact-match linking, the effective coverage estimate was only 16.7% [95% CI: 12.5–18.7%] and 13.9% [95% CI: 10.6–15.2%] for structural quality and process quality adjustment, respectively, which were further less by 1.7% and 1.4% when computed for the inequity-adjusted coverage. The measures were further diminished with ecological linking as linking via administrative boundaries yielded an estimate of 15.9% [95% CI: 12.3–17.8%] for facility readiness adjustment and 12.8% [95% CI: 9.2–14.1%] for process quality adjustment.

Analogously, geographical linking via Euclidean buffers deflated the coverage by 18.1% and 20.5% for accommodating structural and process quality, respectively. Effective coverage for inequity-adjusted crude coverage declined further marginally. Quality-adjusted measures for raster-based linking were found to be less than buffer linking with structural-quality-adjusted coverage of a meagre 14.9% [95% CI: 11.8–19.2%] and process-quality-adjusted estimates of 12.9% [95% CI: 8.5–15.9%]. Similarly, linking via KDE resulted in the lowest effective coverage estimates as coverage dropped by 19.8% and 21.2% post discounting of structural and process quality of care.

#### 3.3.3. Antenatal Care

The proportion of pregnant women receiving full antenatal care from public health facilities was 36.2% [95% CI: 25.1–34.7%]. The effective coverage, by assigning the structural quality score of the exact facility where the visit was conducted was 19.3% [95% CI: 18.6–24.3%]; however, coverage adjustment via process quality yielded an estimate of 26.2% [95% CI: 22.9–29.7%]. The inequity- and quality-adjusted coverages were lower at 15.6% (structural quality) and 21.1% (process quality). Linking of ANC-related visits with the quality of facilities within sub-district boundaries resulted in an effective coverage score of 17.6% [14.2–19.9%] and 23.1% [20.2–29.4%] for structural and process quality adjustment, respectively.

The estimates were marginally greater for linking via Euclidean buffers [17.2%, 95% CI: 12.5–21.4% for structural quality adjustment and 23.6%, 95% CI: 19.4–29.1% for process quality adjustment] and linking via a raster-based travel time grid of 2 h [18.7, CI: 15.2–22.3% for structural quality adjustment and 24.7%, CI: 20.5–29.6% for process quality adjustment]. The deviation in coverage from effective coverage was 12.1% (structural quality) and 5.1% (process quality) with linking via KDE, albeit that this difference was exacerbated to 21.5% (structural-quality) and 15.9% (process quality) for inequity-adjusted contact coverage.

#### 3.3.4. Delivery Care

The utilization of public health facilities for childbirth was high at 90.9% [95% CI: 89.6–98.2%], which declined by 40.8% for the structural-quality-adjusted measure of effective coverage and 26.7% for the process-quality-related measure of effective coverage when delivery-related episodes were linked to the exact provider of care. The inequity-adjusted effective coverage via exact-match linking was 48.1% and 61.6%, respectively. For the entire area of ecological linking, effective coverage estimates via process quality adjustment were higher than structural quality adjustment. The coverage declined to 46.7% [95% CI: 41.8–49.9%] when accounting for the structural quality of care and 55.3% [95% CI: 51.2–60.3%] for process quality adjustments via the administrative boundaries linkage approach. Likewise, inequity-adjusted coverage also exhibited a drop of high magnitude after quality adjustments.

Geographical linking via Euclidean buffers precipitated a decline in coverage estimates by around half for the structural quality measures and around one-third for the process quality measures. The magnitude of plunge in coverage estimates after standardizing for the quality of care was similar for inequity-adjusted coverage. The divide between the crude coverage and adjusted coverage measures was 42% (structural quality) and 28.4% (process quality) with raster-based travel time linking. Finally, KDE-based linking resulted in a structural-quality-adjusted coverage of 48.2% [95% CI: 45.2–52.3%] and process-quality-adjusted coverage of 62.3% [95% CI: 59.7–66.8%].

#### 3.3.5. Postnatal Care

The overall contact coverage of postnatal care in public health facilities was 18.4% [95% CI: 14.2–23.3%]. The quality adjustment led to a diminution in coverage by 9.7% (structural quality) and 6.9% (process quality) and a shrink in inequity-adjusted coverage by 8.9% (structural quality) and 6.3% (process quality) when postnatal visits were linked by the exact source of provider. The effective coverage post structural quality adjustment was 7.9% [95% CI: 5.2–10.1%] and post process quality adjustment was 11.0% [95% CI: 8.2–16.3%] with ecological linking using administrative boundaries.

However, geographical linking using Euclidean buffers culminated in a decrease in coverage levels by 10.3% and 8.3% for adjustment in structural quality and process quality, respectively. Further, the inequity- and structural-quality-adjusted coverage via this approach was 7.4% and 9.2%, respectively. The digression of effective coverage from contact coverage was 9.9% (structural quality) and 7.8% (process quality) when linkage was performed using the travel time grid. Structural-quality-adjusted estimates, however, declined additionally and were reduced to 7.1% [95% CI: 3.9–11.3%] and further to 6.5% for adjustments in inequity along with the quality. Process quality correction, however, resulted in a coverage level of 10.8% [95% CI: 6.8–13.4%] and an estimate of 9.9% for inequity-adjusted coverage.

#### 3.3.6. Inpatient Care

The contact coverage of hospitalization in public health facilities was 78.9% [95% CI: 89.6–98.2%], and inequity-adjusted coverage was 90.7%. Inpatient episodes when linked to the quality scores of exact facilities where hospitalization was sought resulted in a reduction in structural-quality-corrected effective coverage by more than half [33.1%, 95% CI: 29.6–36.7%] and process-quality-corrected effective coverage by half [35.5, 95% CI: 29.8–38.6%]. Invariably, as with other service packages, the ecological linking extenuated the effective coverage estimates of inpatient care. Structural-quality- and process-quality-corrected coverage dwindled by 49.8% and 47.6%, respectively, for administrative linkage.

Congruously, linking via Euclidean buffers contracted the inpatient coverage to 29.9% [95% CI: 26.1–34.2%] for structural quality adjustment and 32.4% [95% CI: 28.1–36.8] for process quality adjustment. Inequity-adjusted coverage nose-dived from 64.7% to 24.5% (structural quality adjustment) and 26.6% (process quality adjustment), respectively. Raster-based travel time linking contracted coverage by 48% for structural quality correction and 45.7% for process quality correction. Lastly, effective coverage upon linking via KDE was deflated to the proportion of 30% [95% CI: 25.8–35.1%] ex-post structural quality adjustment and 33.0% [95% CI: 28.8–36.2%] ex-post process quality adjustment. Inequity-adjusted coverage also declined by 40.1% and 37.6% for structural and process quality adjustments, respectively.

### 3.4. Comparison of Varied Linking Methods

The dissimilitude in effective coverage estimates obtained using varied approaches are depicted in Table 4. The effective coverage estimates generated via the adjustment of structural quality exhibited a significant variation from exact-match linking when linking was performed via raster-based travel time and KDE (4.8% and 5.5%, respectively) for routine immunization. For ambulatory care, KDE linked estimates deviated from exact-match linking by 2.6%, whereas the other linking methods did not vary significantly.

Linking via Euclidean Buffer digressed by 2.1% and 3.8% for antenatal care and delivery care, respectively. Effective coverage for delivery marked a divergence of 3.4% by administrative linking as well. However, estimates derived via all the ecological methods varied significantly from exact-match linking with a difference of 4%, 3.2%, 2.2%, and 3.1% for administrative linking, Euclidean buffer linking, raster-based travel time linking, and KDE linking, respectively. For the effective coverage obtained via process quality adjustment, significant discordance was demonstrated in the estimates computed via geographical linking techniques of Euclidean buffer (3.4%), raster-based travel time (3.6%), and KDE (2.7%) vis-à-vis exact-match linking for routine immunization. The process-quality-adjusted effective coverage metric for ambulatory care was invariant for different linking methods. Administrative boundaries and Euclidean buffer-based estimates varied by 3.1% and 2.6%, respectively, for antenatal care and 8.9% and 3.7% for delivery care. KDE-based estimates for delivery care also deviated from exact-match linking by 1.9%. In attenuation with structural-quality-adjusted effective coverage, the process-quality-adjusted effective coverage estimates computed via all the ecological methods significantly differed from the exact-match approach by magnitudes of 4.2%, 3.1%, 2.3%, and 2.5% for administrative boundary linking, Euclidean buffer linking, raster-based travel time linking, and KDE-based linking, respectively.

Lin’s concordance correlation coefficient (*ρ_C_*) measured pairs of observations obtained via varied ecological linking methods relative to the gold standard measures estimated via exact-match linking. McBride’s decision rule [23] was employed to ascertain the strength of concordance/discordance between various measures. The ecological linking methods via administrative boundary (*ρ_C_* = 0.93) and Euclidean buffers (*ρ_C_* = 0.96) were concordant with the gold standard in mapping routine immunization. In addition to these two ecological linking methods, a level of agreement was also found with linking via raster-based travel time (*ρ_C_* = 0.91) for ambulatory care. However, for antenatal and delivery care, linkage using geo-spatial linking via raster-based travel time (*ρ_C_* = 0.95 and *ρ_C_* = 0.93) and kernel density estimation surfaces (*ρ_C_* = 0.91 and *ρC* = 0.90) exhibited concordance. For agreement, Lin’s concordance correlation coefficient was *ρ_C_* = 0.94, *ρ_C_* = 0.96, *ρ_C_* = 0.98, and *ρ_C_* = 0.90 for linking via administrative boundaries, Euclidean buffers, raster-based travel time, and kernel density estimation surfaces) for post-natal care, thereby demonstrating the reliability of the entire gamut of ecological linking method as a proxy for individual linking.

## 4. Discussion

The study employed a multitude of approaches to estimate the metric of quality-adjusted effective coverage via linking data on need and service contact from population-based surveys with data on service quality from health facility assessment and patient exit surveys. To our knowledge, this is the first study from India to develop a metric of effective coverage by coalescing and linking different datasets using the suite of Geographical Information System (GIS) techniques. Our investigation revealed that most individuals were able to report their specific source(s) of care and the proportion of matching was highest for routine immunization and antenatal care as the entire gamut of care seeking was sought from public providers within the administrative boundaries of the districts for these services. The highest proportion of linking was effectuated for administrative boundaries amongst the ecological approaches, whereas the proportion of population linked plummeted sharply for linking using geographical boundaries such as Euclidean distance buffers and travel time raster. We refined the method by extricating kernel density estimated surfaces by adopting an adaptive bandwidth based on the level of facility and incorporating physical barriers to model the distance–decay effect; however, a smaller proportion of care-seeking episodes were linked using this approach due to the low density and quality score of providers and consequently, lower weight exerted by the providers.

Our findings resonate with and extend the recent empirical work highlighting the complex, bidirectional relationship between health system efficiency and equity in the pursuit of universal health coverage [24]. Evidence from LMICs has suggested that efficiency and equity do not evolve in a fixed sequence; rather, their trajectories are shaped by the institutional and contextual dynamics of individual health systems. This reinforces the need to move beyond linear models of UHC progress and instead adopt measurement frameworks that can capture simultaneity and context-specific interactions. By applying an effective coverage framework that integrates service readiness, travel-time-adjusted access, and patient experience in a fragile and data-scarce subnational setting, our study contributed to the operationalization of such situated measurements. It underscored that efficiency gains and equity improvements are not mutually exclusive goals, but deeply interdependent—requiring integrated metrics and tailored strategies that reflect the structural realities of under-resourced settings.

One of the salient findings of our study was a significant decline in the estimates of effective coverage from crude coverage via both structural quality and process quality adjustment. The dissimilitude between contact coverage and quality-adjusted coverage was present for all the interventions, i.e., routine immunization, ambulatory care, antenatal care, delivery care, postnatal care, and inpatient care for the entire slew of linking approaches viz. exact-match linking and ecological linking via administrative boundaries, Euclidean buffers, raster-based travel time, and kernel-density-estimation-based surfaces. All the services except ambulatory care exhibited a greater plunge in structural-quality-adjusted measures of effective coverage vis-à-vis process-quality-adjusted measures of effective coverage.

Our findings are congruous with previous literature delving into the estimation of effective coverage in low- and middle-income settings. A pilot study conducted in Zambia divulged the effective coverage of the management of childhood illness through exact-match linking to be 15 points lower than crude coverage because of the lower structural quality of providers [19]. Another study from Côte d’Ivoire revealed effective coverage estimates to be 13–63% lower than the care-seeking estimates from a population survey for antenatal care, delivery care, newborn care, postnatal care, and sick child care. Analogous to our findings, this gap was more for structural-quality-adjusted measures than process-quality-adjusted measures [25]. Further, adding quality content to contacts, three settings in Nigeria, Ethiopia, and India linked household surveys and frontline worker/skilled birth attendant assessment to discern the slump in quality-corrected coverage levels for antenatal care, delivery care, postpartum, and postnatal check-up for children. A declivity of 50%, 52%, and 69% (Nigeria, Ethiopia, and India, respectively) for antenatal care; 9%, 8%, and 39% (Nigeria, Ethiopia, and India, respectively) for skilled-birth delivery; 7%, 3%, and 54% (Nigeria, Ethiopia, and India, respectively) for post-partum care and 4%, 3%, and 19% (Nigeria, Ethiopia, and India, respectively) for post-natal care was observed while jumping the cascade from contact coverage to high-quality coverage [6]. The cross-country evidence also corroborates very low levels of effective coverage across eight African nations of Haiti, Kenya, Malawi, Namibia, Rwanda, Senegal, Tanzania, and Uganda, where quality-corrected coverage averaged 28% for antenatal care, 26% for family planning, and 21% for sick child care [15]. Most of the literature, however, was tethered to investigations of maternal and child health outcomes, whereas our study extended the dimensions to encompass other essential service packages as well such as ambulatory care and inpatient care in general.

Our study encompassed only those episodes where care was being sought from public providers, whereas care sought from private providers was excluded from the study. The rationale of assessing only public providers stemmed from the fact that in our study setting, an enormous 92% of the population resided in the rural and remote areas, where the presence of formal private providers was negligible. Most of the individuals in rural areas sought private care either from the informal or unlicensed providers, for whom there is considerable evidence of variable quality which is generally unaligned with clinical standards and lack of accountability mechanisms [5,26,27] or from the exiguous formal private providers in district centres which were outside their service environment (defined by distance and time). The informal providers were excluded from the study as there is clear evidence from LMICs that the quality of services provided by informal providers is suboptimal. Including such care in effective coverage calculations, without rigorous quality adjustment, may introduce further bias due to the unmeasured variability in care quality and data reliability, which can falsely inflate the coverage estimates and obscure the true extent of health system performance gaps. Further, a few studies have also suggested that the private sector is broadly ineffectual in advancing financial protection against ill health in an LMIC setting [28]. Additionally, informal providers pose a formidable challenge with regard to the monitoring and quality assurance. The quality-adjusted effective coverage metrics are most policy relevant when tied to services that are amenable to public sector improvement and routine monitoring [29]. Given the absence of reliable data and the challenges in standardizing the quality indicators for informal providers, their inclusion could confound rather than clarify the service delivery gaps. Conjointly, there is a dearth of information on the type, size, and utilization rates of the informal provider sector in resource-limited settings [30].

The analysis was restricted to the public sector to maintain both internal validity and policy relevance, given that public services are the primary locus of state accountability and the most feasible target for quality improvement interventions at scale [31]. Also, since the rationale of our study was to assess the quality-adjusted coverage which is amenable to the policy change and routine monitoring, the private providers predominated by the informal providers were excluded from the study. This exclusion aligns with the best practices in LMIC health systems research, which often prioritizes traceable and regulatable sources of care [32]. However, our study does not dismiss the role of private or informal providers, but rather acknowledges the methodological and ethical challenges of integrating them into routine metrics in settings where regulatory oversight is minimal and quality measurement infeasible. As health systems evolve, future work must explore innovative strategies to capture and improve care hybrid metrics and quality across all sectors, particularly through regulation, standardization, and engagement with pluralistic health systems.

The findings indicated that process-quality-adjusted coverage was greater than the structural-quality-adjusted coverage for services such as immunization, maternal, and inpatient care. Conversely, the process quality measure was less than the structural quality measure for ambulatory care. In India, traditionally amongst the three categories of Donabedian’s measures of the quality of healthcare (structure, process, and outcomes), structural measures have been emphasized more vis-à-vis other measures [5]. However, in the last few years, the Government of India launched some initiatives such as the National Health Mission to address the process quality of care as well, particularly for maternal and child care. As a result of these interventions and regular monitoring, in our study area, the care processes for some services improved significantly, although the capacity and readiness of the public health facilities in terms of the infrastructure, equipment, and drug availability remained limited. On the other hand, the care processes for ambulatory care were found to be affected by the irregular facility timings, staff absenteeism, and inadequate staff training. Furthermore, the other evidence from the Indian setting is also suggestive of the poor process quality of ambulatory care stemming from the irregular presence and clinical competency of the providers, particularly in the lower-tier facilities [33].

Our study advances the field by integrating structural and process quality through the triangulation of household, health facility, and patient-experience data. Notably, it introduced methodological innovations that directly address the long-standing limitations in the literature. These include the use of raster-based travel time modelling adjusted for topography and physical barriers, incorporation of distance–decay functions to reflect facility catchment dynamics, and kernel density estimation to account for differential provider pull—a significant refinement over conventional vector-based or administrative linkage methods [6,34]. Furthermore, the application of mixed-methods and geospatial linkage techniques in a high-vulnerability, data-scarce setting demonstrates that such approaches are not only feasible but essential for revealing intra-district heterogeneity and health system bottlenecks.

Although the study was conducted in a fragile and geographically remote setting, this context offers critical insights into the functioning of health systems under systemic constraints. Rather than limiting the external validity, the region’s characteristics—service fragmentation, infrastructure deficits, and access barriers—mirror the structural inequities present across many underserved geographies in low- and middle-income countries (LMICs), including rural, peri-urban, and post-conflict areas [17]. Thus, the setting serves as a meaningful stress test for methodological innovations in estimating effective coverage. Through this work, we attempted to propose a scalable, transferable framework for linking household and provider data that accommodates the limitations of routine health information systems. The integration of multiple linking methods—ranging from exact-match to ecological techniques including administrative boundaries, travel-time surfaces, and kernel density estimation—demonstrates methodological versatility and responds to a growing imperative for flexible measurement strategies where direct linkage is infeasible [34,35]. By explicitly testing sensitivity and concordance across approaches, our study advances the empirical basis for linking need, utilization, and quality in effective coverage metrics [1,6]. Moreover, the incorporation of spatial decay functions, physical barriers, and adaptive bandwidths enhances the realism and portability of our methods, offering applicability across heterogeneous health system configurations.

## 5. Policy Recommendations

Based on the findings and context, various demand and supply-side recommendations can be propounded to augment the various quality measures. India has enforced a multitude of interventions for quality promotion such as Indian Public Health Standards (IPHS) 2008, National Quality Assurance Standards for Public Health Facilities (N-QAS) 2020, Mera Aspataal (My hospital) 2016, LaQshya (Labour room Quality Improvement Initiative) 2017, and the National Patient Safety Implementation framework (2018–2025). However, the persistent and pervasive non-compliance of these protocols and guidelines rendered the quality assurance ineffectual. Hence, the monitoring and regular assessment of quality checklists with an exhaustive matrix to capture all the aspects of the quality of care is recommended at regular intervals to gauge the level and progress in quality parameters for prompt redressal. Further, augmentation of another marker of health system performance entailing accountability and governance is recommended. Hybrid forms of accountability with a multi-stakeholder approach inviting external members into internal mechanisms, with an amalgamation of vertical (external mechanisms primarily involving citizens and communities) and horizontal (internal mechanisms between different branches of government or through specifically constituted organizations for the same) accountability is being suggested in India [36], which is relevant to our setting as well.

In order to bolster the structural quality of care, initiatives such as the upgradation of 1,50,000 public healthcare facilities into Health and Wellness Centres (AB-HWCs) to deliver comprehensive primary healthcare was launched under the national flagship Ayushman Bharat Program in 2018 [37,38]. The scheme envisaged the expansion of a range of services much beyond maternal and child care services to encompass NCDs, palliative and rehabilitative care, oral, eye and ENT care, mental health, and first-level care for emergencies and trauma, including free essential drugs and diagnostic services. AB-HWCs were also conceptualized to implement the e-Sanjeevani platform of the Ministry of Health to provide patient-to-doctor outpatient and doctor-to-doctor teleconsultation services using the Hub and Spokes model, where medical colleges and district hospitals are set up as dedicated Hubs and Health and Wellness Centres function as Spokes. However, the roll-out of the scheme which was planned in a phased manner has been lackadaisical and only one-third of centres have been operationalized nearly two years after the promulgation of the scheme; therefore, it is recommended to expedite the operationalization of AB-HWCs to augment the quality of primary healthcare in public facilities.

In India and other low- and middle-income countries, the literature is predominantly focused on structural constraints. Nonetheless, a poor quality of care in low-resource settings is also attributed to a lack of incentives in health system/information problems in the healthcare market, combined with a lack of accountability amongst providers and poorly functioning governance institutions in health systems [5]. For example, in our study area, health workers (most of whom were absorbed and retained as contractual workers under the National Health Mission for the last 15 years and serving “last mile” communities) were sensitive to inadequate and intermittent remuneration, poor working conditions, and poor supervision. As a corollary, many of the health workers were engaged in “dual practice”, pilfering public goods (drugs and diagnostics), and absenteeism. Hence, to mitigate the impact of low remuneration, financial incentives such as remote location and border area allowance in conjunction with improved accommodation facilities in isolated areas need to be introduced. Also, impeding the demand for the regularization of contractual doctors and paramedical staff causing disgruntlement and dissatisfaction amongst workers, and hence, a poor quality of care should be addressed on a priority basis.

Our findings also revealed the lack of supervision and monitoring of health workers, especially in peripheral, remote, and border health facilities and absence of comprehensive Monitoring and Evaluation (M&E) framework and guidelines. Several state governments in India have undertaken quality improvement initiatives (mostly focused on maternal and child health) combined with independent evaluations of performance and the impact of these initiatives. The states of Bihar and Uttar Pradesh in India with the worst health indicators collaborated with external donors and researchers to implement strategies such as nurse mentoring and the direct observation of deliveries where trained observers were recruited to both impart training and monitor the reproductive, maternal, new born, and child health. Such interventions culminated in the improvement of the quality of both healthcare facilities and health workers. For example, in Bihar, a significantly higher proportion of women at mentored PHCs received the recommended clinical care as compared with the women at non-mentored PHCs, and the overall total score of the quality of care, in terms of percent of tasks performed, increased by 14% with the training of health workers [39]. Similarly, the mentoring intervention in rural and poor districts of Karnataka led to improved clinical practices, the improved availability of supplies and equipment, and strengthened referral processes. A Cluster Randomized trial in the region established that the intervention improved facility readiness and provider preparedness in managing institutional births and associated complications during childbirth [40]. Such an intervention intending to upgrade the process quality of service delivery can prove to be propitious in our setting. In conjunction with health workers’ mentoring, we prescribe a more inclusive Participatory Monitoring and Evaluation (PM&E) with a community praxis approach which is central to reflect the perspectives and aspirations of those directly affected by enabling the engagement and empowerment of the primary stakeholders.

Another pertinent policy intervention of mHealth targeting Community Health Workers (CHWs) such as ASHA (Accredited Social Health Activists) workers and AWWs (Anganwadi workers) to improve their performance via increased access to information, work planning, and decision support reducing the barriers to information should be considered. In LMICs, the multitude of digital health interventions defined under m-health have incorporated various functions ranging from client education and behaviour change communication sensors, point of care diagnostics vital registration, data collection and reporting, electronic health records, electronic decision support, provider-to-provider communication, provider work planning and scheduling, training and education, HR management, training and education, supply chain management and financial transactions, and incentives [41]. A pilot of such mHealth interventions in Bihar on ASHA and AWWs suggested an increase in the proportion of beneficiaries receiving visits from workers at different life stages and improved practices related to maternal and newborn health [42]. Further, another enquiry into the cost-effectiveness of m-health interventions from rural Uttar Pradesh revealed that these interventions were cost-effective from a societal perspective [43]. Thus, the scaling up of these m-health interventions in the resource constraint setting in our study area is recommended for improved quality via community health workers.

## 6. Strengths and Limitations

Our study has attempted to bridge the shortcomings of the previous literature pertaining to the linking of various surveys to estimate effective coverage. First, our study was conducted at a subnational level employing robust sampling techniques, thus producing valid and reliable estimates which were representative at the district level and amenable to policy interventions. Second, a census of public health facilities was conducted subsuming the facilities at all the levels of hierarchical structure, vis-à-vis a selection of sample introducing misclassification bias in the previous literature. Third, the quality of spatial data used in the analysis was evaluated based on (a) positional accuracy, (b) thematic accuracy, (c) temporal accuracy, (d) completeness, (e) logical consistency, and (f) usability. Fourth, provider assessment to compute quality scores was conducted based on both the objective criterion of structural quality of facilities investigated via the availability of tracer items for the provisioning of services and subjective criterion of patients’ satisfaction and perception upon receiving care. Fifth, a population survey and provider assessment were conducted concomitantly in the same timeframe, ensuring temporal accuracy. Sixth, linking was conducted using both direct and indirect methods including many geospatial techniques that capture the service environment in the defined geographical area, as linking to the nearest provider alone introduces many biases due to circumventing the lower-level facilities by care seekers. Seventh, external influences such as geographical and topographical impediments to access care were incorporated by conducting the linkage using raster-based travel time and augmented KDE-based estimated surfaces, allowing for physical barriers. Eighth, the misclassification bias emanating from the cluster displacement in previous works employing DHS datasets was absent in our study as geospatial coordinates were not displaced while performing linkage.

Our study, although employing a synoptic and robust methodological approach to discern the quality-adjusted effective coverage is underscored by certain limitations. First, there was an exclusion of private and informal providers from the provider assessment, thereby underestimating the overall coverage levels and overestimating the provider score in the service environment. Second, due to the pervasive bypassing of facilities, individuals do not always seek care from their nearest provider or one that is designated to serve the area/service environment, and in fact travel longer distances to seek disproportionately more care from higher-level providers and providers with better quality/availability of resources, thus interjecting a self-selection bias that can lead to the distortion of the quality-adjusted scores, especially for estimated scores via KDE linkage. Third, the population in need and contact coverage was ascertained by self-reporting of individuals which is susceptible to recall bias, and over-reporting of care seeking from public providers vis-à-vis traditional/faith-based healers due to social desirability bias. *Fourth*, quality scores generated by subjective measures can be inflated due to the possibility of the Hawthorne effect while interviewing exit clients. Fifth, some caveats emanated from the geographic linking approach, capping the maximum distance/travel time, resulting in a high proportion of unlinked episodes due to the lower provider density in remote areas and consequently, under-estimation/over-estimation of quality-adjusted estimates as an average of quality score by provider type, which was then ascribed to unlinked episodes. Sixth, the study cannot be generalized to the national level with a diverse healthcare provider landscape for which further large-scale studies should be conducted to evaluate the feasibility of linking methodology. However, our study estimated a rather succinct metric with profound policy implications, and can be scaled/replicated at the subnational level and in areas with a low variation in preparedness within the provider category and less heterogeneity in the provider mix to seek care.

## 7. Conclusions

A significantly steep decline was found along the cascade from crude coverage to effective coverage via varied linking approaches. The drop was found to be more pronounced when crude coverage was adjusted for structural quality vis-à-vis the process quality of care. Overall, the effective coverage and inequity-adjusted effective coverage of a comprehensive set of healthcare interventions across maternal and child, and ambulatory and inpatient care was 36.4% and 33.3%, respectively. The composite metric of effective coverage was the lowest for postnatal care (10.1%) and highest for immunization care (78.7%). A significant divergence ranging from −2.1 to −5.5 for structural quality and −1.9 to −8.9 for process quality was exhibited between exact-match linking and ecological linking. The abysmal quality of care was underscored as the major barrier to healthcare coverage in the study area. Thus, policy recommendations targeted towards health systems’ strengthening and quality improvement such as the effective implementation of government flagship programs, strengthening of referral linkages, and introduction of performance-linked incentive schemes to attract and retain workforce and community-based monitoring are suggested.

## Figures and Tables

**Table 1:** Proportion of care-seeking episodes linked to public health facilities from reported source of care category by linking method.

Linking Method	Service Type					
	Routine Immunization	Ambulatory Care	Antenatal Care	Delivery Care	Postnatal Care	Inpatient Care
1. Exact-match Linking	100	91.5	100	96.9	96.9	92.6
**2. Ecological Linking**						
**2.1. Administrative Linking**						
Linking via Sub-District Boundaries	100	100	100	100	100	100
**2.2. Geographical Linking**						
2.2.1. Euclidean Buffers Linking	91	88	86	75	84	86
2.2.2. Raster-based Travel Time Linking	40	39	39	30	39	28
2.2.3. Kernel Density Estimates Linking	62	48	31	31	31	27

**Table 2:** Crude coverage, concentration indices and inequity-adjusted coverage of care-seeking episodes within district by facility level.

	Hospitals			Primary Health Centres			Subcentres			Overall		
	Coverage	HI	Inequity Adjusted	Coverage	HI	Inequity Adjusted	Coverage	HI	Inequity Adjusted	Coverage	HI	Inequity Adjusted
Immunization	15.4 [12.1–19.7]	0.04	14.8	17.4 [14.1–22.6]	0.03	16.9	61.6 [57.4–64.6]	0.0	61.6	94.5 [89.6–98.2]	0.04	90.7
Ambulatory	19.0 [14.6–21.8]	0.09	17.3	8.0 [5.4–12.6]	0.03	7.76	7.0 [4.3–11.8]	0.02	6.86	33.9 [30.2–38.4]	0.05	32.2
Antenatal	18.9 [15.2–23.6]	0.23	14.5	8.6 [5.7–11.4]	0.01	8.51	8.8 [4.9–12.3]	−0.07	9.41	36.2 [25.1–34.7]	0.19	29.3
Delivery	71.2 [67.7–74.2]	0.12	62.6	10.6 [7.5–14.8]	−0.02	10.8	9.0 [5.7–12.8]	−0.04	9.36	90.9 [84.2–93.8]	0.04	87.3
Postnatal	8.5 [5.3–11.5]	0.17	7.05	5.6 [3.1–9.3]	0.09	5.09	4.3 [2.4–6.5]	−0.05	4.51	18.4 [14.2–23.3]	0.08	16.9
Inpatient	70.0 [67.2–73.5]	0.23	53.9	8.9 [5.4–13.6]	0.04	8.54	NA	NA	NA	78.9 [73.8–82.4]	0.18	64.7

**Table 3:** Estimates of structural-quality-adjusted and process-quality-adjusted coverage by service type.

	Crude Coverage		Structure-Adjusted Coverage		Process-Adjusted Coverage	
	Crude Coverage	Inequity-Adjusted Coverage	Quality-Adjusted Coverage	Quality- and Inequity-Adjusted Coverage	Quality-Adjusted Coverage	Quality- and Inequity-Adjusted Coverage
**1. Exact-Match Linking**						
Routine Immunization	94.5 [89.6–98.2]	90.7	75.9 [70.3–80.4]	73.6	81.6 [76.2–83.4]	79.2
Ambulatory Care	33.9 [30.2–38.4]	32.2	16.7 [12.5–18.7]	15.0	13.9 [10.6–15.2]	12.5
Antenatal Care	36.2 [25.1–34.7]	29.3	19.3 [18.6–24.3]	15.6	26.2 [22.9–29.7]	21.1
Delivery Care	90.9 [84.2–93.8]	87.3	50.1 [47.4–55.1]	48.1	64.2 [61.2–68.8]	61.6
Post-Natal Care	18.4 [14.2–23.3]	16.9	8.7 [6.2–10.4]	8.0	11.5 [8.7–13.6]	10.6
Inpatient Care	78.9 [73.8–82.4]	64.7	33.1 [29.6–36.7]	27.1	35.5 [29.8–38.6]	29.1
**2. Ecological Linking**						
**2.1 Administrative Linking**						
**Linking via Sub-District Boundaries**						
Routine Immunization	94.5 [89.6–98.2]	90.7	74.1 [69.8–78.1]	71.1	80.4 [77.2–84.1]	77.2
Ambulatory Care	33.9 [30.2–38.4]	32.2	15.9 [12.3–17.8]	15.1	12.8 [9.2–14.1]	12.2
Antenatal Care	30.2 [25.1–34.7]	36.2	17.6 [14.2–19.9]	14.2	23.1 [20.2–29.4]	18.7
Delivery Care	90.9 [84.2–93.8]	80.0	46.7 [41.8–49.9]	44.8	55.3 [51.2–60.3]	53.1
Post-Natal Care	18.4 [14.2–23.3]	12.3	7.9 [5.2–10.1]	7.3	11.0 [8.2–16.3]	10.1
Inpatient Care	78.9 [73.8–82.4]	64.7	29.1 [25.2–32.1]	23.9	31.3 [26.8–34.2]	25.7
**2.2 Geographical Linking**						
**2.2.1 Linking via Euclidean Buffers**						
Routine Immunization	94.5 [89.6–98.2]	90.7	75.0 [70.3–78.7]	72.0	78.2 [75.3–82.5]	75.1
Ambulatory Care	33.9 [30.2–38.4]	32.2	15.8 [11.4–18.6]	15.0	13.4 [10.3–18.2]	12.7
Antenatal Care	30.2 [25.1–34.7]	36.2	17.2 [12.5–21.4]	13.9	23.6 [19.4–29.1]	19.1
Delivery Care	90.9 [84.2–93.8]	80.0	46.3 [41.3–50.4]	44.4	60.5 [57.2–63.8]	58.1
Post-Natal Care	18.4 [14.2–23.3]	12.3	8.1 [5.0–10.9]	7.4	10.1 [7.2–14.9]	9.2
Inpatient Care	78.9 [73.8–82.4]	64.7	29.9 [26.1–34.2]	24.5	32.4 [28.1–36.8]	26.6
**2.2.2 Linking via Raster-Based Travel Time**						
Routine Immunization	94.5 [89.6–98.2]	90.7	71.1 [66.7–74.2]	68.2	78.0 [74.2–83.4]	74.9
Ambulatory Care	33.9 [30.2–38.4]	32.2	14.9 [11.8–19.2]	14.1	12.9 [8.5–15.9]	12.2
Antenatal Care	30.2 [25.1–34.7]	36.2	18.7 [15.2–22.3]	15.1	24.7 [20.5–29.6]	20.0
Delivery Care	90.9 [84.2–93.8]	80.0	48.9 [45.3–52.8]	46.9	62.5 [58.2–66.8]	60
Post-Natal Care	18.4 [14.2–23.3]	12.3	8.5 [6.1–10.3]	7.8	10.6 [7.1–14.5]	9.7
Inpatient Care	78.9 [73.8–82.4]	64.7	30.9 [27.4–34.5]	25.3	33.2 [28.4–36.1]	27.2
**2.2.3 Linking via Kernel Density Estimates**						
Routine Immunization	94.5 [89.6–98.2]	90.7	70.4 [66.2–73.8]	67.6	78.9 [75.3–82.4]	75.7
Ambulatory Care	33.9 [30.2–38.4]	32.2	14.1 [10.2–19.7]	13.4	12.7 [9.8–15.7]	12.1
Antenatal Care	30.2 [25.1–34.7]	36.2	18.1 [14.4–22.5]	14.7	25.1 [22.8–29.7]	20.3
Delivery Care	90.9 [84.2–93.8]	80.0	48.2 [45.2–52.3]	46.3	62.3 [59.7–66.8]	59.8
Post-Natal Care	18.4 [14.2–23.3]	12.3	7.1 [3.9–11.3]	6.5	10.8 [6.8–13.4]	9.9
Inpatient Care	78.9 [73.8–82.4]	64.7	30.0 [25.8–35.1]	24.6	33.0 [28.8–36.2]	27.1
**Confidence Interval in Parenthesis**						

**Table 4:** Absolute and relative differences between exact-match and ecological linking methods.

	Routine Immunization		Ambulatory Care		Antenatal Care		Delivery Care		Postnatal Care		Inpatient Care	
Structural-Quality-Adjusted Coverage												
	Abs.	Rel.	Abs.	Rel.	Abs.	Rel.	Abs.	Rel.	Abs.	Rel.	Abs.	Rel.
Exact-Match Linking	Reference		Reference		Reference		Reference		Reference		Reference	
Administrative Boundary Linking	−1.8	−2.37	−0.8	−4.79	−1.7	−8.81	−3.4 *	−6.79	−0.8	−9.20	−4 *	−12.08
Euclidean Buffer Linking	−0.9	−1.19	−0.9	−5.39	−2.1 *	−10.88	−3.8 *	−7.58	−0.6	−6.90	−3.2 *	−9.67
Raster-Based Travel Time Linking	−4.8 *	−6.32	−1.8	−10.78	−0.6	−3.11	−1.2	−2.40	−0.2	−2.30	−2.2 *	−6.65
Kernel Density Estimate Linking	−5.5 *	−7.25	−2.6 *	−15.57	−1.2	−6.22	−1.9	−3.79	−1.6	−18.39	−3.1 *	−9.37
**Process-Quality-Adjusted Coverage**												
	**Abs.**	**Rel.**	**Abs.**	**Rel.**	**Abs.**	**Rel.**	**Abs.**	**Rel.**	**Abs.**	**Rel.**	**Abs.**	**Rel.**
Exact Match Linking	Reference		Reference		Reference		Reference		Reference		Reference	
Administrative Boundary Linking	−1.2	−1.47	−1.1	−7.91	−3.1 *	−11.83	−8.9 *	−13.86	−0.5	−4.35	−4.2 *	−11.83
Euclidean Buffer Linking	−3.4 *	−4.17	−0.5	−3.60	−2.6 *	−9.92	−3.7 *	−5.76	−1.4	−12.17	−3.1 *	−8.73
Raster-Based Travel Time Linking	−3.6 *	−4.41	−1	−7.19	−1.5	−5.73	−1.7	−2.65	−0.9	−7.83	−2.3 *	−6.48
Kernel Density Estimate Linking	−2.7 *	−3.31	−1.2	−8.63	−1.1	−4.20	−1.9 *	−2.96	−0.7	−6.09	−2.5 *	−7.04

* statistically significant at 95%.

## Data Availability

The authors conducted the field study to generate the dataset. The primary dataset generated and analyzed is provided in the Supplementary File. A more detailed dataset used in the study can be made available from the corresponding author upon reasonable request.

## Supplementary Materials

The following supporting information can be downloaded at: https://www.mdpi.com/article/10.3390/ijerph22040561/s1, Table S1: Study participants, service coverage indicators, and source of data; Table S2: Exemplar information on tracer items collected from health facility assessment; Table S3: Exemplar of information on tracer items collected from patient exit surveys. Further, background and details on geographical linking via kernel density estimates is provided in the Supplementary File.
