# Supplementary material for: Linking Household and Service Provisioning Assessments to Estimate a Metric of Effective Health Coverage: A Metric for Monitoring Universal Health Coverage
Source: Int J Environ Res Public Health. 2025 Apr 3;22(4):561. doi: 10.3390/ijerph22040561 (PMC12027280; doi:10.3390/ijerph22040561)
Supplement: Supplementary file 1 [file ijerph-22-00561-s001.zip › ijerph-3494069-supplementary.pdf]

Supplementary Materials

Table S1. Study participants, service coverage indicators and source of data.

Service Coverage Indicator	Target Population	Definition	Source
Routine Immunization	Children aged 1–2 years with facility issued immunization card	Proportion of children aged 12-23 months who received full recommended immunization (OPV- 1, 2, 3; DPT-1, 2, 3; BCG, and Measles) in the last 365 days prior to survey.	Household Survey
Ambulatory Care	Individuals reporting ailments in the last 30 days prior to survey	Proportion of ailing individuals who sought care for ailment from any level of facility	Household Survey
Adequate Antenatal Care	Women aged 15–49 years who had delivered in last 365 days prior to survey	Proportion of pregnant women receiving adequate ANC care (at least 4 ANC visits, ≥100 IFA tablets and 2 doses of Tetanus Toxoid	Household Survey
Delivery Care	Women aged 15–49 years who had delivered in last 365 days prior to survey	Proportion of pregnant women delivering in any level of institutional facility.	Household Survey
Adequate Postnatal Care	Women aged 15–49 years who had delivered in last 365 days prior to survey	Proportion of pregnant women receiving postpartum checkup within 48 hours of delivery and 6 ASHA visits up-to 42 days after delivery	Household Survey
Inpatient Care	Individuals requiring hospitalization in the last 365 days prior to survey	Proportion of individuals requiring inpatient services admitted to any level of facility in the last 365 days.	Household Survey

Table S2. Exemplar information on tracer items collected from Health Facility Assessment.

Amenities	Composite score of tracer indicators capturing mean availability of amenities and basic infrastructure such as Adequate Premises, Power Supply With Backup, Adequate Rooms And Area Of Building, Waiting Room, Seating Arrangement, Residential Accommodation, Immunization/Family Planning/Counselling Area, Clean Source Of Drinking Water, Adequate Clean Toilets, Registration Counter, Blood Storage Facility, Functional Ambulance And Other Referral Transport For Emergency, Dispensing Cum Drug Store Room, Communication Channels Like NIC Terminal , Email etc.
Equipment	Composite score of tracer indicators capturing mean availability of functional basic equipment such as ***a)*** Weighing Scale, Stadiometer, BMI Charts, BP Instrument, Stethoscope, Thermometers for ***Routine Care b)*** Labor Table, Delivery Kit For Normal, Assisted And Vacuum Delivery, Suction Machine, Autoclave/Sterilizer, IV Stand, Adult Resuscitation, MVA/MTP Suction Aspirators etc. for ***Delivery Care c)*** Weighing Machine For Infant, Neonatal Resuscitation Bag With Mask, Cord Ties Radiant Warmer, Phototherapy Unit, Neonatal Laryngoscope And Endotracheal Intubation Tubes, Mucus Extractors With Suction Tube And Feeding Tubes etc. for ***Neonatal Care d)*** Vaccine Carrier, Cold Box, ILR (Large), ILR (Small), Deep Freezer etc. for ***Immunization Care e)*** IUD Insertion Kits for ***Reproductive Care f)*** Standard Surgical Set, Steam Sterilizer Drum, Facility For Oxygen Administration, Boyles Apparatus, Ventilator, Defibrillator, Functional Blood Bag Refrigerators, Anesthesia Machines, Blood Storage Facility, Pulse Oximeters, Oxygen Cylinders And Fumigation Apparatus etc. for ***Inpatient/Surgical Care.***
Medicines	Composite score of tracer indicators capturing mean availability of drugs and consumables such as ***a)*** ORS, IFA, Vitamin A, Antiallergics, Antibiotics, Povidone Iodine, Hypodermic needle and syringe, Absorbent Cotton and Gauge etc. for ***Routine Care b)*** Emergency Drug Tray, Nifedipine, Labetalol, Digoxin, Dicycloamine, Hyoscine Butyle Bromide, Doxycycline Hydrochloride, Gentamycin Sulphate, Ampicillin Trihydrate, Declofenac, Sensorcrain, Oxytocin, Sodium Lactate etc. for **Delivery Care** ***c)*** Injectibles like Adrenaline Tartrate, Amikacin, Aminophyline, Calcium Gluconate, Dopamine, Gentamycin Sulphate, Dextrose Anhydrous, Phenobarbitone Sodium, Potassium Chloride etc. for **Neonatal Care** ***d)*** Emergency Contraceptive Pills, Nirodhs/Condoms, Oral Pills, IUCD's for ***Reproductive Care e)*** BCG, DPT, Measles, OPV, TT etc. for ***Immunization Care f)*** Other Miscellaneous Drugs for treatment Of NCDs such as drugs for TB, CVDs, Respiratory Diseases, Antidiabetics, Diauretics etc. ***g) Consumables*** such as Plain and Floyes Catheter, Chromium Catgut, Mersilk, Intra Cath Cannulas, surgical spirit, Urobag etc. for ***Inpatient/Surgical Care.***
Diagnostics	Composite score of tracer indicators capturing mean availability of **a)** ***Diagnostic tests*** such as Hb test, Urine test, Stool analysis, Albumin test, Pregnancy test, Malaria test, TB test , ECG, X ray, Stress test, LTF, Ultrasound, CT scan, RPR for syphilis, Colposcopy, Memography, Spirometry and other serological tests such as Coomb's test, WIDAL test and ELISA etc ***b) Laboratory reagents*** such as Colorimeter, Carbol Fuchin, Methylene blue, Sulphuric acid, Phenol/hyperchlorite, Immersion oil, Methylated spirit, Crystal violet stain, Acetone ethanol decolorizing solution, Safaranine stain, ABO and Rh antibodies, KOH solution for Whiff test and Gram's iodine ***c) Consumables & kits*** such as Sputum containers, Peripheral blood smear for detection in case of fever, Pregnancy testing kits, Urine albumin and sugar testing kits, Sputum containers, Glasswares-test tubes, pippetes, glass-rods, glass-slides, coverslips etc.<br>***d) Instruments*** such as Microscope, Centrifuge and Semi-Autolyzer etc.
Infection Control Protocols	Composite score of tracer indicators capturing mean availability of **a)** ***Infrastructure*** such as Deep burial pit for waste management, Sewage system, Handwashing facility etc. and ***b) Adherence of protocols*** such as **Asepsis** (sterilization, disinfection, cleaning twice a day, fumigation after each procedure) **Waste disposal** (color-coded bins/bags, puncture proof containers for disposing needles and sharps) **Infection control practices** (segregation and treatment of bio-medical waste, use of disposable syringes and examination gloves, use of masks and protective clothes including cap, gown, gloves, hand washing with soaps, sanitizer etc.)

Guidelines	Composite score of observed mean availability of guidelines such as ***a)*** Integrated Disease Surveillance Programme, Standard Operating Procedures in National Quality Assurance System Policy for ***Routine Care b)*** Operational Guidelines for Antenatal Care and Skilled Attendance at Birth by ANMs/SNs/LHVs or SBA, Handbook for ANMs/SNs/LHVs as SBA, SBA quality protocol posters, Handbook on Preconception & Prenatal Diagnostic Technique Act, 1994 ,Guidelines for JSSK, JSY, Maternal and Newborn Health Toolkit etc. for ***Delivery Care c)*** Operational Guidelines for facility based integrated management of neonatal and childhood illness, Navjaat Shishu Suraksha Karyakram- Basic Newborn Care and Resuscitation Program- Training Manual and Facilitator's Guide for ***Neonatal Care d)*** Immunization Infokits for health workers, ASHAs and Anganwadi workers, Routine Immunization monitoring formats, Handbook for Vaccine and Cold chain handlers, Immunization Handbooks for Medical Officers and Health Workers, National Vaccine Policy, Routine Immunization monitoring formats etc. for ***Immunization Care e)*** Standard Operating Procedures for Sterilization in camps, Contraceptive update Manual for doctors and facilitator's guide, Reference Manual on Minilap and Emergency Contraceptive Pill, Handbook for RMNCH/FP Counselors etc. for ***Reproductive Care f)*** Standard Operating Procedures for Inpatient Management, Surgical Safety Checklist in Operation Theatres etc. for ***Inpatient***/***Surgical Care***.
Trained Staff	Composite score of observed mean availability of staff as per sanctioned strength such as ***a)*** Multipurpose workers, Pharmacist, Laboratory Assistant and Medical Officer for ***Routine Care b***) Gynecologist, Medical Officer trained in Emergency and Obstetric Care, MTP/MVA, Skilled Birth Attendant, Nurse/Midwife(regular), Nurse/midwife(contractual), Round clock nurse/LHV/ANM trained in SBA, RMNCH+A, MCTS etc. for ***Delivery Care*** **c)** Medical Officer and other para-medical staff trained in NSSK, IMNCI for ***Neonatal Care d)*** Medical and paramedical staff trained in Immunization and Cold Chain for ***Immunization Care e)*** Medical Officer trained in Non-Scalpel Vasectomy, IUD insertion etc. for ***Reproductive Care*** and ***f)*** General Surgeon, Anesthetist and other Specialists for ***Inpatient***/***Surgical Care.***

Table S3. Exemplar of information on tracer items collected from Patient Exit Surveys.

Domain	Components
Interpersonal	Composite score capturing elements of patient-provider interaction viz. a) Behaviour of doctor and other health workers b) Willingness of health workers to interact with patient c) Explaining the results in local language d) Maintaining confidentiality and privacy of patients and e) Non-discriminatory behavior of health workers.
Affordability	Composite score capturing elements of paying for health services viz. a) Cost incurred for user fees and package cost b) Cost incurred for drugs and diagnostics c) Catastrophic payment incurred during visit/hospitalization d) Distressed financing by borrowing/selling of assets.
Accessibility	Composite score capturing elements of geographical access to health services viz. a) Distance from Facility b) Travel time to facility c) Availability of motorable transportation d) Referral transportation
Availability	Composite score capturing elements of physical resources viz. a) Availability of doctor b) Availability of paramedical staff c) Availability of prescribed drugs in the facility d) Availability of diagnostics.
Technical Quality	Composite score capturing elements of competence of providers and adherence to quality standards viz. a) Information provided on diagnosis b) Adequate time spent with patients c) Conducted diagnostics d) Information provided on dosage e) Information provided on side-effects/contradictions/precautions etc.
Physical Environment	Composite score capturing elements of setting in which care is delivered viz. a) Amenities like drinking water, functional toilet b) Waiting area with seating arrangement c) Cleanliness of ward and toilets d) Infection control protocols in wards and laboratories etc.
Efficacy	Composite score capturing elements of desired/intended experiences of provider encounter viz. a) Quickness of staff b) Length of time spent on visit c) Length of time spent waiting for service provider d) Financial risk protection/subsidy received.
Continuity	Composite score capturing elements regarding the trust and decision to continue care with same provider viz. a) Decision to visit provider again b) Recommend provider/facility to others c) Decision to continue care with referred provider/facility

2.3.2.2. Geographical Linking

Linking via Kernel Density Estimates

KDE is a spatial technique that distributes a discrete point value over a continuous surface. Through this approach, weighted pull of the providers was accounted to reflect the level of draw which health facilities exert over the households within their catchment area as a source of care for ailment. There are myriad forces exerting pressure on treatment seeking such as a) Type of provider b) Distance to provider and c) Provider Readiness and Preparedness. KDE through its approach, enables the radius and intensity of pull exerted by health facilities to vary via incorporating Distance Decay Function, depending upon the characteristics of provider. The service area of a facility is analogous to a geographical territory within which it can be construed, that benefits obtained from the facility are greater than the costs of accessing it. As distance from the facility increases, the costs necessary to overcome that impediment increases, reducing the net benefit of attending the facility. This technique assumes that each facility serves a specific catchment area and that the draw on the population to those services decreases with increasing distance

from the facility(1). The KDE is more robust over other methods as following characteristics are embedded in this approach: a) Providing an estimate of density in a continuous surface which is unrestrained by administrative boundaries, and thus, explicating a better representation of spread of people and services across landscape b) Embodying a decaying effect of distance on facility's service area and c) Examining an additive service environment with the conjecture that a high service environment from multiple facilities has greater effect than a high service environment from single facility (2).

The weighting of distance of care-seeking episodes from a particular provider, x, can be expressed mathematically as follows (3):

$$f(x) = \frac{1}{nh}\sum_{i=1}^{n} k\left(\frac{x - x_i}{h}\right) \quad (1)$$

where n is the number of observations in the sample, h is the bandwidth or the smoothing parameter, $k(x)$ is the defined kernel function and $(x - x_i)$ represents the Euclidean distance between each point i and the provider location. The Kernel bandwidth/search radius (h) can be computed as:

$$Search\ Radius = 0.9 * \min\left(SD, \sqrt{\frac{1}{\ln(2)} * D_m}\right) * n^{-0.2} \quad (2)$$

where, D_m is the (weighted) median distance from (weighted) mean center, n is the number of points if no population field is used, or if population field is supplied, n is sum of population field values, SD is the standard distance.

Parameter Selection

Kernel density estimation requires user-specified kernel size and probability density distribution. Therefore, linking via KDE entails specification of certain parameters i.e., a) Kernel bandwidth/ Kernel size b) Density variable which determines the probability density distribution across the kernel and c) Grid size. The output of KDE is contingent on the choice of these parameters, and it is pertinent to select an appropriate specification. Firstly, with regards to kernel size, a larger kernel captures the phenomenon across an expansive area, but larger bounds are susceptible to patterns becoming too generalized, however, smaller kernels can delineate the localized patterns but can stymie the larger patterns. Thus, a varied bandwidth was specified with higher order facilities (District Hospital and Community Health Centers) receiving a larger kernel size vis-a-vis peripheral facilities (Primary Health Centers and Sub Centers), explicated as below:

a) District Hospital and Community Health Centers—10 km
b) Primary Health Centers—5 km
c) Sub-center—2 km

The provider Quality Score (structural and process) was specified as Density/Population value, modelling stronger pull/draw within their catchment area for providers with better quality scores. Gaussian/normal distribution assigning greater weight closer to the center and lesser to the parameters was used in the study. Distance from the center, hence, is thus purported to have a diminishing influence as distance from the facility increases, the lesser influence it exerts. Finally, a Grid Size of 100 meters was set to extricate output faster. Augmenting the methodology used in previous studies, KDE incorporating Physical Barriers such as waterbodies and snow-capped areas etc. were employed in this study. There are two-fold ways in which presence of barriers alters the computation of density a) Either increasing the distance between the feature and the cell

where density is being calculated b) Or excluding a feature from calculation. The Kernel density operation with a barrier produces estimated surfaces which are more realistic and accurate.

References

1. Do M, Micah A, Brondi L, Campbell H, Marchant T, Eisele T, et al. Linking household and facility data for better coverage measures in reproductive, maternal, newborn, and child health care: Systematic review. *J. Glob. Health [Internet]* **2016** Sep 10 [cited 2021 Feb 18];6(2). Available from: www.jogh.org
2. Spencer J, Angeles G. Kernel density estimation as a technique for assessing availability of health services in Nicaragua. *Health Serv Outcomes Res Methodol [Internet].* **2007** Dec 6 [cited 2021 Jan 9];7(3–4):145–57. Available from: https://link.springer.com/article/10.1007/s10742-007-0022-7
3. Silverman BW. Density Estimation for Statistics and Data Analysis Chapter 1 and 2 [Internet]. 2003 [cited 2021 Jan 9]. Available from: https://books.google.com/books?hl=en&lr=&id=e-xsrjsL7WkC&oi=fnd&pg=PA1&ots=iyKkmp_I-l&sig=BKG23ZQZLuySVHXw2xV8Q9t3ixo
4. Skiles MP, Burgert CR, Curtis SL, Spencer J. Geographically linking population and facility surveys: Methodological considerations. *Popul. Health Metr. [Internet].* **2013** Aug 8 [cited 2021 Jan 5];11(1):14. Available from: https://pophealthmetrics.biomedcentral.com/articles/10.1186/1478-7954-11-14
5. Carter ED, Ndhlovu M, Eisele TP, Nkhama E, Katz J, Munos M. Evaluation of methods for linking household and health care provider data to estimate effective coverage of management of child illness: Results of a pilot study in Southern Province, Zambia. *J. Glob. Health* **2018**, *8*.
